# Exploring the Gut-Brain Axis for the Control of CNS Inflammatory Demyelination: Immunomodulation by *Bacteroides fragilis’* Polysaccharide A

**DOI:** 10.3389/fimmu.2021.662807

**Published:** 2021-05-05

**Authors:** Deniz Erturk-Hasdemir, Javier Ochoa-Repáraz, Dennis L. Kasper, Lloyd H. Kasper

**Affiliations:** ^1^ Department of Immunology, Harvard Medical School, Boston, MA, United States; ^2^ Department of Biology, Eastern Washington University, Cheney, WA, United States; ^3^ Department of Microbiology and Immunology, Geisel School of Medicine at Dartmouth College, Hanover, NH, United States

**Keywords:** immunomodulation, microbiota, EAE (experimental autoimmune encephalomyelitis), multiple sclerosis, symbiotic molecules, *Bacteroides fragilis*, polysaccharide A (PSA)

## Abstract

The symbiotic relationship between animals and their resident microorganisms has profound effects on host immunity. The human microbiota comprises bacteria that reside in the gastrointestinal tract and are involved in a range of inflammatory and autoimmune diseases. The gut microbiota’s immunomodulatory effects extend to extraintestinal tissues, including the central nervous system (CNS). Specific symbiotic antigens responsible for inducing immunoregulation have been isolated from different bacterial species. Polysaccharide A (PSA) of *Bacteroides fragilis* is an archetypical molecule for host-microbiota interactions. Studies have shown that PSA has beneficial effects in experimental disease models, including experimental autoimmune encephalomyelitis (EAE), the most widely used animal model for multiple sclerosis (MS). Furthermore, *in vitro* stimulation with PSA promotes an immunomodulatory phenotype in human T cells isolated from healthy and MS donors. In this review, we discuss the current understanding of the interactions between gut microbiota and the host in the context of CNS inflammatory demyelination, the immunomodulatory roles of gut symbionts. More specifically, we also discuss the immunomodulatory effects of *B. fragilis* PSA in the gut-brain axis and its therapeutic potential in MS. Elucidation of the molecular mechanisms responsible for the microbiota’s impact on host physiology offers tremendous promise for discovering new therapies.

## Introduction

Mammals have co-evolved with eons of resident microorganisms that play an integral role in regulating the host immunity (1). These microorganisms live in a complex community called microbiota, which is dominated by bacteria and includes archaea, fungi, and viruses (2). Analysis of the composition and the human microbiota’s diversity has significantly improved by culture-independent methods and next-generation sequencing (3, 4). An updated catalog based on a metagenomic assembly of microbiomes across world populations shows that over 150,000 bacterial genomes can be found in the human body (5). The gastrointestinal tract harbors most of these microbes, some producing immunomodulatory molecules to educate the host immune system (6). The composition and gut microbiota function can affect the susceptibility to and progression of a wide range of diseases in the intestine and the extraintestinal tissues such as the central nervous system (CNS) (7, 8). While several microbial species have been identified concerning specific pathologies in humans, the mechanistic understanding of how the symbiotic molecules interact with the host is still limited. Determining the molecular mechanisms of the microbial molecules is crucial for the development of prophylactic and therapeutic interventions. *B. fragilis* polysaccharide A (PSA) is a prototypical symbiotic antigen that has been invaluable for understanding the mechanisms directing microbiota–host interactions (9). PSA mediates gut homeostasis by directing cellular and physical development of the immune system (10), stimulating Tregs (11) *via* plasmacytoid dendritic cells (PDCs) (12), and protecting animals from experimental diseases like colitis (11, 12), asthma (13), or pulmonary inflammation (14), and experimental autoimmune encephalomyelitis (EAE) (15–17). EAE is an animal model of multiple sclerosis (MS), an inflammatory demyelinating CNS disease (18). MS is characterized by inflammation and axonal damage resulting in progressive disability due to neurodegeneration. Although the disease’s etiology is not entirely understood, multiple genetic and environmental factors have been implicated in MS’s onset and progression. Mounting evidence suggests that microbiota plays an essential role in the development of the disease (19).

In this review, we cover some of the most recent literature on the gut-brain axis in the context of CNS inflammatory demyelination. Because of the impact of gut microbes regulating the immune system and the immune-mediated responses that characterize EAE/MS, we hypothesize that the large pool of microbes and microbial products present in the gut is an excellent source of novel therapeutics. Furthermore, we propose that PSA produced by *B. fragilis* is an identified symbiont factor model for immunomodulation. PSA could be one of the possibly numerous bacterial cellular components capable of promoting protective responses against neuroinflammation. Accordingly, our review will summarize how PSA regulates immune responses in MS/EAE and discusses PSA’s therapeutic potential.

## Gut Microbiota

Although utero colonization is still debated (20), it is known that microbial colonization starts mainly after birth (21). It is suggested that establishing a diverse and balanced microbiota in early life is essential for developing a healthy immune system (22, 23). An imbalance in the microbiota’s composition and function (dysbiosis) during this window of opportunity can have long-lasting consequences later in life, causing a wide range of immune diseases (24). The microbial composition of the infant’s gut depends on many factors (25), including the type of delivery (26), gestational age (27), antibiotic use (28), and the mode of feeding (29). Human milk oligosaccharides (HMOs) in breast milk promote *beneficial* microbes like *Bifidobacterium* species in breast-fed infants (30). Cessation of breast-feeding and introducing solid foods drives the infant’s gut microbiome’s maturation (31), gradually reaching an adult-like composition after three years of life (32). Gut microbiota in adulthood is dominated by Bacteroidetes and Firmicutes and includes Actinobacteria, Proteobacteria, Verrucomicrobia, archaea, viruses, fungi, and protozoa (33, 34). While the gut microbiota in adults is more stable than in infants, the specific microbial species can vary interpersonally, creating a unique composition for every individual.

In homeostatic conditions, the host and the microbiota benefit each other and coexist in a mutualistic symbiosis (35). The host offers a nutrient-rich environment for the microbiota. In return, the microbiota provides metabolites (36), vitamins (37), and other micronutrients to the host by fermenting undigested dietary components in the large intestine (38). The microbiota’s ability to produce energy by digesting complex carbohydrates in the gut has been an evolutionary driving force for establishing the host-microbiota symbiotic relationship (39, 40). In addition, the gut microbiota participates in the development of the host immune system and balances defense and tolerance to maintain homeostasis (41).

While the use of fecal microbiota transplantation (FMT) as a treatment for gastrointestinal problems in Chinese medicine dates back to the 4^th^ century (42), the interconnectedness of gut microbiota, CNS, and neuropsychiatric health is a concept from the early 19^th^ century (43). It is now known that the fundamental impact of the microbiota on host physiology reaches far outside the gastrointestinal tract and extends to CNS. Recently it is proposed that there is a bidirectional relationship between the gut microbiota and the CNS (44, 45). This reciprocal interaction occurs through different routes involving endocrine, immune, and neural mechanisms. The function of microbiota in the gut-brain axis is believed to affect the etiology of a wide range of neuropsychiatric disorders, including Parkinson’s disease (46, 47), Alzheimer’s disease (48, 49), depression (50, 51), anxiety (52, 53), autism (54, 55), amyotrophic lateral sclerosis (56) and multiple sclerosis (57–66). Increasing evidence points to alterations in the gut microbiota composition in patients with neuropsychiatric disorders. However, the molecular mechanisms by which the microbiota modulates these diseases are not fully understood. A mechanistic understanding of microbiota’s role in the gut–brain axis will help develop prophylactic and therapeutic interventions for CNS diseases that are increasingly affecting large populations.

## Gut Microbiota and CNS Inflammatory Demyelination

Multiple Sclerosis (MS) is an immune-mediated debilitating disease initiated by the immune system attacking the neuron protecting myelin sheet, which results in inflammation, chronic demyelination, axonal degeneration, and loss of brain volume (67, 68). MS affects around 400,000 people in the United States (69) and 2.5 million worldwide, mainly living in higher latitudes (70). MS is divided into four clinical types: Relapsing-Remitting (RR-MS), Secondary Progressive (SP-MS), Progressive Relapsing (PR-MS), and Primary Progressive (PP-MS), with the majority of patients suffering from RR-MS type. There are various environmental risk factors associated with disease onset and progression (vitamin D, latitude, viral infections, smoking, diet) and genetic disposition (71). The immunopathology of MS is mainly driven by inflammatory CD4^+^ T cell responses characterized by an increase in Th1 and Th17 cells (72) and decreased or impairment in Treg cells (73). Increasing importance is now appreciated for B cells’ role in both the progression and modulation of this condition (74). CD20^+^ B cells are targeted with monoclonal antibodies as approved MS therapies (75). Cerebrospinal fluid (CSF) of MS patients contains oligoclonal bands produced by plasmablasts and plasma cells (76), some of them auto-reactive against myelin self-peptides (77). B cells are present in CSF, CNS parenchyma, and meninges of MS patients, and germinal centers were identified in MS CNS (78). A recent study of single-cell RNA sequencing in CSF and blood suggest that in MS patients, B cells are clonally expanded and show an active inflammatory signature with memory plasma cell or plasmablast phenotype (79). More significant to this review’s context, IgA produced by B cells that cross-react with gut microbes have been identified in the CNS of MS patients with active lesions (80) and regulate neuroinflammation through IL-10 production (81), highlighting the importance of B cells on the gut-microbiota-brain axis. In addition to T and B cells, dendritic cells and CD8^+^ T cells also play a role in modifying MS’s pathology (82).

Gut microbiota is considered an environmental factor that can promote protective roles in the development of multiple sclerosis. Other environmental risk factors for MS, such as diet, vitamin D, or geography, can directly affect the microbiota’s composition. Gut microbiota can activate immune cells in the intestine or secrete immunomodulatory molecules and metabolites that orchestrate immune responses in the gut-brain axis. Previous studies suggest a link between changes in the composition of gut microbiota and MS pathogenesis. Limited but accumulating evidence points to an altered microbiota in MS patients compared to healthy individuals (57–66). The MS patients’ microbiota shows a decreased abundance of *Bacteroides*, *Parabacteroides*, *Prevotella*, and *Lactobacillus* genera and increased *Akkermansia Blautia*, *Ruminococcus*, and *Bifidobacterium* (83). The relative abundances of members of the domain Archaea, such as *Methanobrevibacter*, are also increased in MS patients’ gut and the bacteria such as *Akkermansia*, while *Butyricimonas* is decreased when compared to healthy controls (63). Recent findings link immunoglobulin (Ig) A (IgA)-coated gut microbiota with MS. IgA is the major neutralizing Ig in the human mucosa, including the gut, but is also found in circulation and periphery and a recent paper reports elevated IgA levels in cerebrospinal fluid of MS patients suffering active neuroinflammation (80). IgA^+^ B cells capable of recognizing gut microbiota are present within active CNS lesions, where elevated *IL-10* transcripts are observed. Previous findings from the same group indicate that IL-10 was a principal component of gut-derived plasma cells’ immunoregulatory role against neuroinflammation (81). There is now increasing evidence for the gut microbiota’s role in the associated neuroinflammatory condition, neuromyelitis optica syndrome (84).

Despite the limitations of any experimental model of disease, EAE mice offer a practical approach to elucidate the clinical relevance of gut microbes in neuroinflammatory disorders, such as MS. The oral administration of broad-spectrum antibiotics reduces the severity of EAE (85–88). The treatment of C57BL/6 EAE mice with kanamycin, colistin, and vancomycin induced protection mediated by invariant natural killer cells T (iNKT) cells associated with reduced production of proinflammatory cytokines (IFN-γ, TNF-α, IL-6, and IL-17) in draining lymph nodes (LN) and a reduction in the percentages of mesenteric LN Th17 cells. In the mesenteric LNs of antibiotics-treated EAE mice, the levels of proinflammatory cytokines were reduced while IL-10 was increased (86). The treatment of SJL/J EAE mice with vancomycin, metronidazole, ampicillin, and neomycin induced protection against the disease that was associated with a reduced production of proinflammatory cytokines (IFN-γ, IL-17) in draining lymph nodes and increased frequencies of Foxp3 expressing CD25^+^CD4^+^T cells and increased levels of IL-10 and IL-13 (85). The ablation of CD25-expressing cells resulted in the lack of protection with antibiotics, while the adoptive transfer of CD25^+^CD4^+^ T cells with enhanced expression of Foxp3 isolated from antibiotics-treated SJL/J mice reduced the severity of EAE in recipient mice (85). The observation that antibiotics given orally but not intraperitoneally can protect the animals from disease emphasizes gut microbiota’s importance in EAE pathophysiology. The results obtained after oral versus intraperitoneal administration of ampicillin supported these findings (87). A significant reduction in inflammatory antigen-presenting cells (peripheral macrophages and resident microglia) was also observed and associated with the protective effects promoted by antibiotics against EAE (88). Mechanistically, the protective effects induced by the oral treatment with antibiotics could be associated with alterations in microbial populations capable of triggering molecular mimicry pathways towards autoimmunity (87). Studies in GF mice confirmed the impact of the presence of gut microbiota in the severity of EAE. In GF conditions, mice show reduced EAE severity and reduced peripheral proinflammatory signals (89, 90). The causative association between the gut microbiota and EAE remains to be elucidated. EAE induction promotes alterations in the composition of the gut microbiota at early phases of disease (44), and disease results in alterations in the intestinal permeability and intestinal proinflammatory responses (91).

The use of gnotobiotic mice in EAE studies helped identify a few essential bacteria for positively or negatively modulating the disease outcome. When GF mice are monocolonized with *Segmented Filamentous Bacteria* (*SFB*), an increase in Th17 cell-mediated responses correlate with exacerbated EAE severity (89). When the MS patients’ microbiota was transferred to germ-free mice, it caused more severe symptoms in the EAE (57) and spontaneous brain autoimmunity (58). Furthermore, fecal microbiota transplantation (FMT) is reported to alleviate disease symptoms in EAE mice (92) and MS patients (93–95). Although it is still elusive if alterations in microbiota’s composition and function are the cause or the result of the disease, microbiome-based therapeutics offer promise for MS treatment.

It has been shown that *Prevotella histicola* suppresses EAE through Tregs (96), while *Lactobacillus reuteri* exacerbates the disease through pathogenic CD4^+^ and CD8^+^ T cell responses (97). The prophylactic administration of individual lactobacilli strains reduces EAE severity through diminished myelin oligodendrocyte glycoprotein (MOG)- T cell reactivity (98). In contrast, the treatment with a mixture of three strains (*Lactobacillus paracasei* DSM 13434, *Lactobacillus plantarum* DSM 15312, and *Lactobacillus plantarum* DSM 15313) was able to reduce the progression of established severe EAE in a mechanism mediated by IL-10-producing Tregs (98). Single species of Enterococci, *Escherichia coli*, and others have also shown promising EAE study results (**Table 1**). The EAE model has successfully addressed the protective role of probiotic formulations with multiple species (105), some of which have already been assessed in MS patients (**Table 1**). A systematic review of the use of probiotic formulations in EAE and MS studies has been recently published (111).

**Table 1:** Single probiotic species and probiotic multi-species mixes evaluated for protection in murine EAE ^1^ and MS clinical studies.

Model of EAE/MS study	Probiotic strains	Primary mechanisms of action proposed	Ref.
Prophylactic, in C57BL/6 EAE	*Escherichia coli Nissle*	Anti-inflammatory effects, reduction of Th1/Th17. Restored intestinal barrier disruption	(99)
C57BL/6 EAE, during disease	*Lactobacillus reuteri*	Reduction of Th1/Th17, reduced proliferation of autoreactive cells, restored dysbiosis	(100)
HLA-DR3.DQ8 double transgenic EAE	*Prevotella histicola*	Increased Treg, anti-inflammatory effects, and reduction of Th1/Th17	(96, 101)
Prophylactic and therapeutic in C57BL/6 EAE	*Lactobacillus paracasei* DSM 13434, *L. plantarum* DSM 15312, and DSM 15313	Increased Treg, anti-inflammatory effects, and reduction of Th1/Th17	(98)
Therapeutic, in C57BL/6 EAE	*Bifidobacterium animalis* and *Lactobacillus plantarum*	Increased Treg, anti-inflammatory effects, and reduction of Th1/Th17	(102)
Wistar rats EAE, during the disease	*Enterococcus faecium* L-3	Increased T cell function, with proposed involvement of IL-10	(103)
Prophylactic, in C57BL/6 and SJL/J EAE	*Pediococcus acidilactici*	IL-10-producing regulatory Tr1 cells	(104)
Therapeutic, in C57BL/6 EAE	Lactibiane iki ^2^	Increased Treg	(105)
Therapeutic, in Theiler’s murine encephalomyelitis virus	Vivomixx ^3^	Anti-inflammatory responses, reduces astrogliosis, increased Bregs	(106)
EAE in Lewis rats	*Lactobacillus plantarum* NCIB 8826 and *L. murines* CNRZ	Reduce cumulative disease burden: Mechanism of action not evaluated	(107)
Prophylactic treatment in C57BL/6 EAE; ongoing EAE.	IRT5 ^4^	Increased IL-10 producing CD4^+^ T cells and IL-producing CD11c^+^ monocytes	(108)
MS; RR-MS subjects on glatiramer acetate *vs*. untreated and healthy controls	Vivomixx	Reduced peripheral monocyte-mediated responses and APC function	(109)
MS; Randomized, double-blind, placebo-controlled trial	*L. acidophilus*, *L. casei*, *L. fermentum*, *Bifidobacterium bifidum*	Improved EDSS, anti-inflammatory effects	(110)

^1^ EAE protection studies performed with PSA and PSA-producing B. fragilis were not included since they are extensively discussed in the manuscript’s body.

^2^ Lactibiane iki: Bifidobacterium lactis LA 304, Lactobacillus acidophilus LA 201, and L. salivarius LA 302.

^3^ Vivomixx: Lactobacillus acidophilus DSM 24735, L. plantarum DSM 24730, L. paracasei DSM 24733, L. delbrueckii subsp. Bulgaricus DSM 24734, Bifidobacterium longum DSM 24736, B. breve DSM 24732, B. infantis DSM 24737, and Streptococcus thermophilus DSM 24731.

^4^ IRT5: Lactobacillus casei, L. acidophilus, L. reuteri, Bifidobacterium bifidum, and Streptococcus thermophilus.

As discussed above, gut microbiota modulates CNS inflammatory demyelination in murine models. In the following sections, we discuss *Bacteroides fragilis*, and its capsular polysaccharide A (PSA), identified as a member of the gut microbiota and microbial product with immunomodulatory effects that we hypothesize can regulate the extent of neuroinflammatory mechanisms associated with CNS demyelinating diseases.

## The Gut Microbiota as a Source for Immunomodulatory Factors: Polysaccharide A (PSA) Produced by *Bacteroides Fragilis*

*Bacteroides fragilis (B. fragilis)*, a prominent species of the genus *Bacteroides* within the Gram-negative Bacteroidetes phylum is part of the normal microbiota of the human colon. Bacteroides species are among the most abundant and adept colonizers of the human gut due to their ability to efficiently utilize complex host and dietary glycans (112) and express different surface structures by phase variation (113). *B. fragilis*, an obligate anaerobic Gram-negative bacillus, colonizes the majority of healthy individuals (114) and has profound effects on host physiology (115). *B. fragilis* was initially identified as the most common anaerobe in clinical isolates from abscesses caused by abdominal trauma and bacteremia (116). When contained in the gut, *B. fragilis* plays an intricate role in the colon and develops a beneficial relationship with the host (9). Monocolonization of germ-free mice with *B. fragilis* leads to the immune system’s cellular and physical development (10, 117, 118). *B. fragilis* can also alleviate intestinal inflammation in animal models of colitis (12, 117, 119) and confer protection against infections (120–124). Recent studies show that *B. fragilis* exerts its beneficiary effects not only locally in the intestine but also systemically in extraintestinal tissues (13, 14, 125, 126) including CNS (15, 54, 127).

The capsular polysaccharide structure of *B. fragilis* plays an essential role in establishing a symbiotic relationship with its host (128). A large part of the *B. fragilis* genome is allocated to enzymes that degrade dietary polysaccharides and produce capsular polysaccharides of the organism (129–132). *B. fragilis* produces eight different distinct capsular polysaccharides, regulated by phase variation at the promoter region (133, 134). Variable expression of polysaccharides A through H creates a remarkable surface diversity which is vital for symbiosis (135, 136) and immunomodulation (112, 137). PSA is most abundantly expressed among those polysaccharides, and its immunomodulatory properties are most extensively studied (138).

PSA is a zwitterionic immunomodulatory polysaccharide consisting of a tetrasaccharide repeating unit (139, 140). The zwitterionic structure with a negative and a positive charge in each repeating unit is essential for the immunological potency of PSA (141, 142) and is required to activate T cells through the major histocompatibility complex II (MHCII) pathway (143, 144). High-resolution LC-MS/MS analysis shows that the terminal-reducing end of PSA contains a covalently attached lipid moiety required to activate antigen-presenting cells and protect against EAE (17). PSA is processed by antigen-presenting cells (APCs) through depolymerization in endocytic compartments in a nitric oxide-dependent manner (145, 146) and presented through the MHCII pathway to activate T cells (143, 147). The beneficial effects of PSA on the host immune system are manifested through multiple mechanisms. Microbial colonization in the gut is essential for host health. Host-specific microbiota is required for the full maturation of a functional immune system (148). Germ-free mice grown in sterile conditions develop physical and functional defects in their immune system, making them predisposed to infectious and inflammatory diseases (149). Recolonizing germ-free (GF) mice with PSA expressing *B. fragilis* mediates immune system development and can correct germ-free animals’ deficiencies (10). PSA-dependent colonization of *B. fragilis* in a unique mucosal niche in the gut results in the induction of regulatory T cells and suppression of Th17 cells (150). WT *B. fragilis* but not the ΔPSA mutant induces anti-inflammatory CD4^+^ CD45Rb^low^ T cell population (119) and protects animals from the T cell transfer model of experimental colitis. In addition, animals orally treated with pure PSA (119) or PSA containing outer-membrane vesicles (OMVs) from WT *B. fragilis* (151) can protect animals from intestinal inflammation. PSA-mediated immunomodulation requires tolerogenic plasmacytoid dendritic cells (pDCs) (12), which activate a specific set of T cells defined as IL-10–producing CD4^+^CD25^+^Foxp3^+^ Treg cells with an inducible phenotype (11). Innate and adaptive immune responses initiated by PSA require toll-like receptor 2 (TLR2). TLR2 is necessary for inducing the genes (e.g., iNOS, MHCII, and CD86) required for processing and presenting PSA by APCs (152). As a result, PSA exposure of APCs increases the antigen presentation capacity of the cells by increasing the expression of MHCII and costimulatory signals, including ICOSL (12). The enhanced production of IL-10 triggered by pDCs after PSA recognition is ablated in the absence of ICOSL/ICOS signal (12). In addition, TLR2 expression on APCs is necessary to induce IL-10 producing CD4^+^ T cells (12) and protection against colitis (11, 12) and EAE (16, 17). PSA is recognized by the TLR2/TLR1 heterodimer in collaboration with Dectin-1 initiating a signaling cascade that involves the phosphoinositide 3-kinase (PI3K) pathway (17). Activation of PI3K pathway leads to phosphorylation and inactivation of glycogen synthase kinase 3β (GSK3β), promoting cAMP response element-binding protein (CREB)-dependent transcription of anti-inflammatory genes. Furthermore, TLR2 directs the expansion of CD39^+^CD4^+^ T cells in response to PSA, required for PSA-mediated protection against EAE. PSA’s EAE protection is ablated in TLR2 (16, 17), TLR1, and Dectin-1 deficient mice (17).

**Figure 1:**
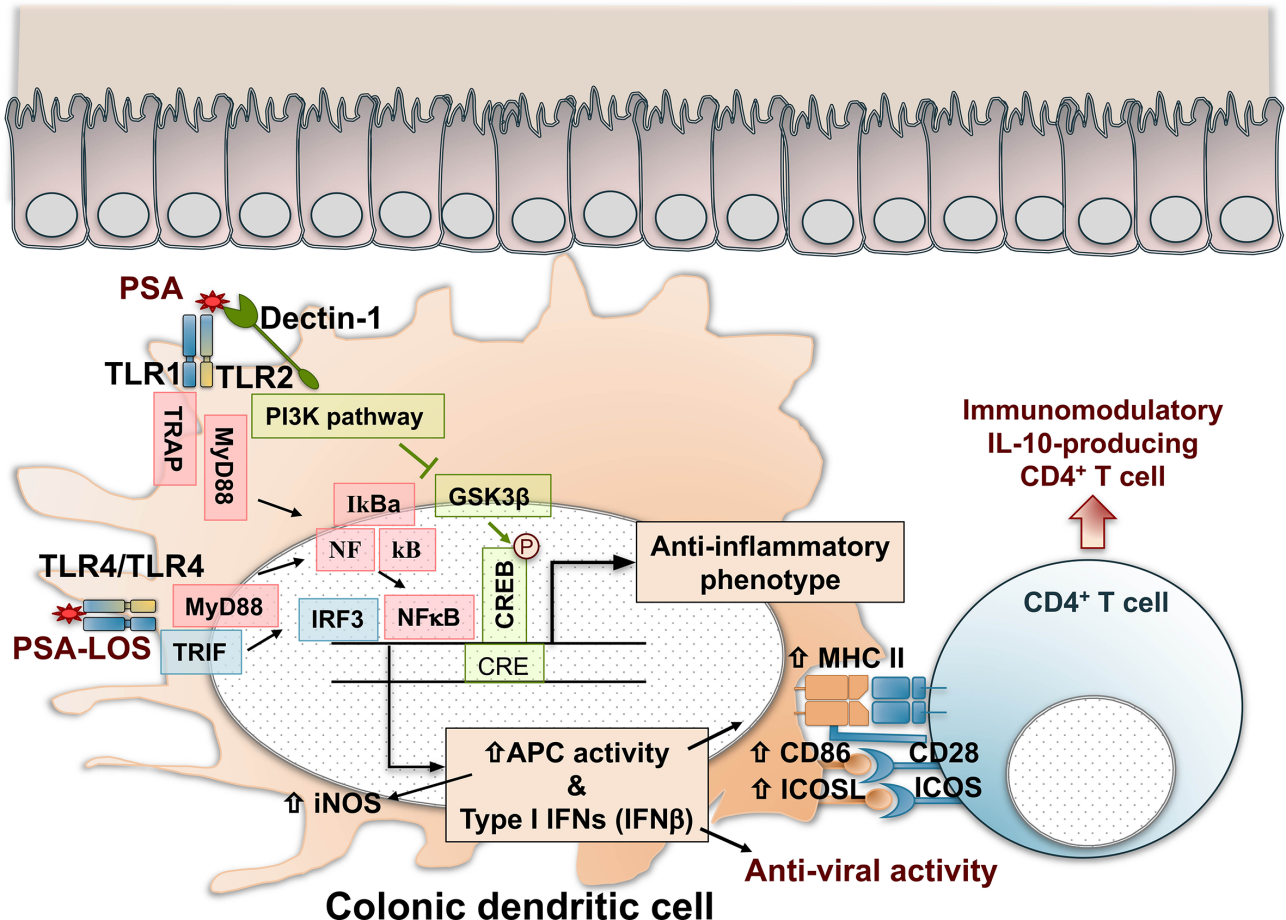
Recognition, cell signaling, and immunomodulatory pathways triggered by PSA in colonic dendritic cells. PSA is recognized by TLR1/TLR2 dimers that result in NF-kB nuclear translocation and IRF-mediated activation of Type I IFN gene expression and enhanced antigen processing and presentation by increased expression of iNOS, MHC class II molecules, and costimulatory signals mediated by CD86 and ICOSL. In addition, Dectin-1, a C-Type Lectin pattern recognition receptor, contributes with TLR2 in the cell signal activation through the PI3K pathway, resulting in the nuclear phosphorylation and activation of CREB, triggering the expression of anti-inflammatory genes. As a result, naïve CD4^+^ T cells are activated and differentiated in IL-10-producing immunomodulatory cells with Foxp3, CD39, Tr1 phenotypes that might depend on the inflammatory condition (IBD, asthma, EAE, or other). PSA recognition by TLR4 dimers induces the production of IFN-β with anti-viral activity through a MyD88 and TRIF-dependent pathway. The activation of TLR4/TRIF is dependent on the lipooligosaccharide (LOS) portion of the polysaccharide. CREB, cAMP response element–binding protein; GSK3β, glycogen synthase kinase 3β; IFN, interferon; IL-10, interleukin 10; iNOS, inducible nitric oxide synthase; IRF, interferon regulatory factors; MHC II, major histocompatibility complex class II; MyD88, myeloid differentiation primary response 88; NFkB, nuclear factor-kB; PSA, polysaccharide A; PI3K, phosphoinositide 3-kinase; TLR, toll-like receptor; TRAP, tumor necrosis factor receptor-associated protein. TRIF, TIR domain-containing adapter-inducing interferon-beta.

The interactions between PSA and the host’s intestinal dendritic cells are multifactorial. PSA recognition by colonic dendritic cells through TLR4-TRIF (TIR domain-containing adapter-inducing interferon-β) domain pathway, through the activation of interferon regulatory factors (IRFs), induces the production of IFN-β with anti-viral activity (153). The activation of the TLR4/TRIF pathway depends on the presence of a lipooligosaccharide (LOS) fraction linked covalently to the polysaccharide, anchoring the macromolecule to the outer membrane of *B. fragilis* (17). PSA produced by *B. fragilis* was identified as a symbiont factor promoting IFN-β-dependent protection against vesicular stomatitis virus infection. PSA’s anti-viral effects were lost in the absence of both TLR4 and IFN-β (153). Thus, the production of IFN-β by colonic dendritic cells with CD103^+^CD11b^-^ and CD103^-^CD11b^+^ phenotypes is regulated by PSA and *B. fragilis* and likely by other commensal microbiota (153). **Figure 1** summarizes the recognition and cellular signaling pathways triggered by PSA in dendritic cells that result in the activation of immunomodulation dominated by IL-10-producing CD4^+^ T cells and anti-viral responses.

## PSA Against Neuroinflammation

Studies demonstrating the impact of *Bacteroides fragilis* and PSA on EAE pathology (15, 127) were the first mechanistic examples of immunomodulation by gut microbiota in multiple sclerosis. *B. fragilis* and PSA’s beneficial effects beyond the gastrointestinal tract are observed most strikingly in the gut-brain axis using EAE. When antibiotic-treated mice were colonized with WT *B. fragilis* but not the ΔPSA mutant, they were protected from EAE (127). Immunoprotection by PSA in EAE requires TLR2, TLR1, and Dectin-1 (17) and a specific tolerogenic DC subset called plasmacytoid dendritic cells (pDC), as shown in **Figure 1** (12). In the EAE model, WT *B. fragilis* protects by inducing Foxp3^+^ Tregs and IL-10, whereas the ΔPSA mutant induces proinflammatory cytokines IL-17 IL-6 and causes pathology. In addition, CD103^+^CD11c^+^ DCs isolated from cervical lymph nodes of ΔPSA-colonized animals are unable to convert Foxp3^-^CD4^+^ T cells into Foxp3^+^Treg cells (127). Furthermore, prophylactic or therapeutic treatment with pure PSA is sufficient to protect animals from EAE (15). PSA can induce accumulation of CD11c^+^CD103^+^ DCs in cervical lymph nodes, which convert naive CD4^+^ T cells into Foxp3^+^ Treg cells (15). IL-10–deficient mice are not protected from EAE, showing that PSA’s immunoprotection in the EAE model requires IL-10–producing Treg cells induced by tolerogenic DCs (15). In addition, PSA-induced regulatory T cells express CD39 independent of their Foxp3 expression and require TLR2 for activation (16). CD39 [nucleoside triphosphate diphosphohydrolase-1 (NTPDase 1)] is an ectoenzyme that degrades ATP released from damaged cells to AMP and adenosine. Previous studies have shown that CD39 is expressed by regulatory T cells (145) and has an essential role in suppressing Th17 cells (154, 155). CD39^+^ Tregs are reduced in MS patients (154), contributing to the Th17-driven pathology in this disease. CD39 deficiency increases IL-17 and decreases IL-10 production in mice and abrogates PSA-induced immunoprotection in EAE (16).

Furthermore, CD39 enhances the migratory capacity of CD4^+^ T cells, which results in PSA-dependent accumulation of CD39^+^ CD4^+^ Foxp3^+^ regulatory T cells in the CNS (156). In the DC-T cell coculture system, using cells from healthy human peripheral blood mononuclear cells (PBMCs), PSA induces IL-10 producing CD39^+^Foxp3^+^ cells *in vitro* (157). Furthermore, PSA increases the expression of CD39 and IL-10 and enhances the suppressive function of Foxp3^+^CD4^+^ cells (157). Similarly, PSA drives differentiation of regulatory T cells and IL-10 production using naïve T cells from MS patients (158). Notably, induced expression of Foxp3 in response to PSA was higher in MS patients than in healthy controls (158).

The protective effects of PSA against neuroinflammation were also addressed in a murine model of viral encephalitis. The induction of neuroinflammation with Herpes Virus was controlled by PSA’s administration that promoted a protective mechanism by IL-10 (126). The phenotypes of IL-10-producing cells induced by the PSA treatment were heterogeneous, with inductions of ICOS^+^CD39^+^CD37^+^CD4^+^ T cells, CD37^+^CD8^+^ T cells, and IL-10-producing B cells. IL-10-producing Tregs were increased in draining LNs of PSA-treated mice compared to PBS-treated mice. The protection against neuroinflammation triggered by the virus was also observed when mice were treated with PSA-producing *B. fragilis* (126). Thus, it appears that PSA is a potent modulator of neuroinflammation, in addition to the protective effects observed against infections (120–124), autoimmunity at the intestinal level (12, 117, 119), asthma (13), or pulmonary inflammation (14). More work is necessary to elucidate whether PSA’s phenotypes depend on the inflammatory pathways triggered in specific target tissues of the different disorders. A recent paper showed that PSA promotes the activation of an interferon responsive gene (IRG) signature responsible for producing inflammatory cytokines and cellular signals resulting in PD1, Lag3, and Tim3 expression (159). PSA regulates Type I interferons’ production by colonic dendritic cells, specifically IFN-β by TLR4-TRIF domain signaling mechanisms (153). The production of IFN-β directs the anti-viral protective responses induced by PSA (153).

## Conclusions

The microbiome research field had expanded continuously since the early 2000s, which helped us understand the human microbiome’s role in health and disease. In recent years, the field’s scope shifted from the characterization of the microbiota composition and its association to diseases to mechanistic and causative understanding of microbes on human health.

The most prominent and most studied effect of gut microbiota is on the immune system, which can influence a wide range of infectious, inflammatory, metabolic, and autoimmune diseases (160). The immune system plays an essential role in the bidirectional communication within the gut-brain axis and modulates diseases in the CNS, including multiple sclerosis. Several different microbial species have been associated with susceptibility to and progression of multiple sclerosis. However, this is still an emerging field, and researchers must address numerous challenges in their research.

Separating correlation from causation in microbiome-disease association studies is one of the biggest challenges in the field. The literature has been dominated by associative studies comparing differences in microbiomes of MS patients with healthy controls. To translate these association studies into the clinic, researchers need to discover which of these differences are causing the disease. In addition, it is challenging to define “healthy microbiota” since there is tremendous intrapersonal and interpersonal variability in the composition of the human microbiota. Dysbiosis referring to disturbances in the microbiota structure is widely used without specific definitions of balanced and imbalanced microbial communities’ compositions. Furthermore, while the GF and gnotobiotic animals are invaluable for microbiome research, animal studies’ applicability to humans needs to be verified.

An accumulating body of research has proven the potential use of gut symbionts as microbial therapeutics. Following the success of FMT in treating *C. difficile* infections, several companies are testing groups of microbes or individual bacteria in clinical trials. However, understanding molecular interactions that shape host-bacterial interactions is crucial for the effective design of microbial therapeutics. This review highlights the microbiota’s role in mediating immune responses to multiple sclerosis, focusing on an archetypical microbial molecule PSA. PSA is a hallmark of symbiotic molecules with immunoregulatory functions. Although further protection and toxicity studies are needed to address PSA’s applicability as a therapeutic, we hypothesize that PSA, and likely other unidentified gut symbiont factors, is a safe and effective alternative for treating multiple sclerosis and other CNS diseases.

## Author Contributions 

DE-H and JO-R contributed equally to the design and preparation of the manuscript, tables, and figures. DK and LK contributed equally to the idea, the design, and the review of the manuscript. All authors contributed to the article and approved the submitted version.

## Conflict of Interest

The authors declare that the research was conducted in the absence of any commercial or financial relationships that could be construed as a potential conflict of interest.
